# A Case of Primary *EGFR* T790M Mutation in Treatment-Naïve Advanced NSCLC: Clinical and Molecular Implications

**DOI:** 10.3390/curroncol33050244

**Published:** 2026-04-24

**Authors:** George Dimitrov, Elitsa Kraevska, Vladislav Nankov, Victoria Hlebarova, Savelina Popovska

**Affiliations:** 1 Department of Medical Oncology, Medical University of Sofia, University Hospital “Tsaritsa Yoanna”, 1527 Sofia, Bulgaria; 2 Centre of Competence in Personalized Medicine, 3D and Telemedicine, Robotic Assisted and Minimally Invasive Surgery—Leonardo da Vinci, 5800 Pleven, Bulgariavladislav.nankov@mu-pleven.bg (V.N.); savelina.popovska@mu-pleven.bg (S.P.); 3 Department of Pathoanatomy, Medical University of Pleven, 5800 Pleven, Bulgaria; 4 Department of Anatomy, Histology, Cytology and Biology, Medical University of Pleven, 5800 Pleven, Bulgaria

**Keywords:** NSCLC, geriatric oncology, oncogene addicted cancer, primary T790M

## Abstract

Background: De novo (pretreatment) *EGFR T790M* mutation is a rare molecular finding in non-small cell lung cancer (NSCLC) and has historically been associated with primary resistance to first- and second-generation EGFR tyrosine kinase inhibitors (TKIs). Evidence guiding optimal first-line management in this subgroup, particularly in elderly patients, remains limited. Case Presentation: We report a case of an elderly patient with treatment-naïve advanced non-squamous NSCLC harboring a concurrent *EGFR* exon 19 deletion and de novo *EGFR T790M* mutation. Given the patient’s age, significant cardiopulmonary comorbidities, and absence of rapidly progressive disease, a multidisciplinary tumor board recommended first-line osimertinib monotherapy. Treatment was well tolerated, with rapid improvement in performance status and no clinically significant adverse events. Serial contrast-enhanced CT restaging demonstrated RECIST 1.1–defined stable disease, without development of new visceral, nodal, cerebral, or osseous metastases. The patient remains on continuous osimertinib therapy with durable disease control at the time of manuscript preparation. Conclusion: Primary *EGFR T790M*–positive NSCLC can achieve durable disease control with first-line osimertinib, even in advanced age. While combination strategies with chemotherapy may improve survival outcomes in selected patients, treatment decisions in elderly individuals must carefully balance efficacy, toxicity, and quality of life. Chronological age alone should not discourage active targeted treatment when guided by molecular profiling and comprehensive clinical assessment.

## Simple Summary

The primary *EGFR T790M* mutation is a rare change in non-small cell lung cancer and is usually associated with resistance to older targeted therapies. There is limited guidance on how to treat patients with this mutation, especially elderly individuals with other health problems. We report a case of an older patient with advanced lung cancer carrying both an *EGFR* exon 19 deletion and a primary T790M mutation. Given the patient’s age and significant heart and lung conditions, the tumor board recommended first-line treatment with osimertinib 80 mg/day alone. The therapy was well tolerated, improved the patient’s daily functioning, and kept the disease stable for several months without serious side effects. This case shows that even frail, older patients can benefit from personalized targeted treatment, highlighting the value of molecular testing and careful clinical assessment to guide therapy.

## 1. Introduction

Non-small cell lung cancer (NSCLC) accounts for approximately 85% of all lung cancer cases worldwide and remains the leading cause of cancer-related mortality globally [1]. Among molecularly defined subtypes, activating mutations in the epidermal growth factor receptor (*EGFR*) gene represent one of the most clinically actionable alterations, occurring in approximately 10–15% of Caucasian and up to 50% of East Asian patients with NSCLC [2]. The most common *EGFR* mutations—exon 19 deletions and exon 21 L858R substitutions—predict sensitivity to EGFR tyrosine kinase inhibitors (TKIs), which have dramatically improved clinical outcomes in this subgroup [3].

A key mechanism of resistance to first- and second-generation EGFR TKIs is the secondary gatekeeper mutation *EGFR T790M* in exon 20, which substitutes methionine for threonine at position 790 and sterically hinders TKI binding [4]. This mutation typically arises as an acquired resistance alteration after initial response to therapy and is detected in approximately 50–60% of patients who progress on first-generation TKIs [5]. However, in rare cases, the T790M mutation can be detected before treatment initiation, referred to as a pretreatment or de novo T790M mutation [6].

The reported frequency of pretreatment T790M varies widely depending on the sensitivity of the assay used. Standard clinical testing platforms such as mass spectrometry, amplification refractory mutation system (ARMS) PCR, or direct sequencing typically identify baseline T790M mutations in only 0.5–2% of *EGFR*-mutant, TKI-naïve NSCLC cases [6,7,8]. With higher-sensitivity techniques such as droplet digital PCR (ddPCR) or MALDI-TOF mass spectrometry, the prevalence increases to 1–8%, and in some ultra-sensitive studies, up to 25–80%, reflecting detection of low-frequency subclones [9,10]. Clinically significant, high-allele-frequency pretreatment T790M mutations remain uncommon, but when present, they are associated with inferior progression-free survival (PFS) and overall survival (OS) compared with T790M-negative *EGFR*-mutant NSCLC, particularly when the variant allele frequency (VAF) exceeds 1% [9,11].

The coexistence of a sensitizing *EGFR* mutation (most often exon 21 L858R or exon 19 deletion) with T790M at diagnosis carries major therapeutic implications. First- and second-generation TKIs, such as erlotinib, gefitinib, and afatinib, demonstrate limited efficacy in these patients due to intrinsic resistance conferred by the T790M mutation [12,13]. In contrast, the third-generation TKI osimertinib is designed to irreversibly inhibit both sensitizing and T790M-resistant *EGFR* mutations, showing robust clinical activity in this setting [14]. Nevertheless, the optimal management of treatment-naïve patients harboring de novo T790M remains uncertain, particularly when accompanied by multiple comorbidities or mixed molecular signals such as concurrent PD-L1 expression.

From a biological standpoint, pretreatment T790M mutations may arise through two mechanisms: (1) low-frequency subclonal events conferring a selective advantage under TKI pressure, or (2) germline mutations associated with hereditary lung cancer predisposition syndromes. Germline *EGFR* T790M carriers represent approximately 50–60% of individuals with high allelic fractions of T790M, often presenting as female never-smokers with lung adenocarcinoma and strong family history [15]. The prevalence of germline *EGFR T790M* in the general population is exceedingly low (~1 in 7500), but genetic counseling is recommended when germline transmission is suspected [16].

We report here a rare case of advanced lung adenocarcinoma harboring a de novo *EGFR T790M* mutation coexisting with an exon 19 deletion, detected before any systemic therapy in an elderly female with severe cardiopulmonary comorbidities and post-COVID pulmonary fibrosis. This case underscores the diagnostic and therapeutic challenges of managing multimorbid patients with uncommon *EGFR* molecular profiles, where standard treatment paradigms may not apply. Furthermore, it contributes to the limited European real-world data on de novo T790M-positive NSCLC and highlights the need for individualized, molecularly guided therapeutic decisions in this distinct subgroup.

## 2. Case Presentation

**Figure 1:**
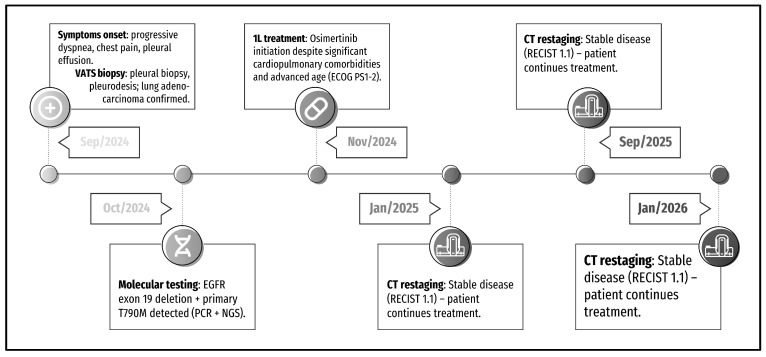
The timeline of main events spans from September 2024 to November 2025.

A 79-year-old Caucasian woman, lifetime nonsmoker, with a significant history of chronic obstructive pulmonary disease (COPD), congestive heart failure, atrial fibrillation, and post–COVID-19 pulmonary fibrosis, presented with progressive exertional dyspnea, pleuritic chest pain, and left-sided pleural effusion. Her past medical history also included systemic hypertension, mitral regurgitation, bronchiectasis, and panlobular emphysema. The patient had been hospitalized in 2021 for severe COVID-19 pneumonia requiring mechanical ventilation and developed chronic interstitial changes thereafter. At presentation in September 2024, she reported increasing shortness of breath at rest and dull thoracic pain unrelieved by standard analgesia. Her Eastern Cooperative Oncology Group (ECOG) performance status was 2. A concise timeline of the case can be seen in Figure 1 and baseline laboratory values in Supplementary Tables S1–S7.

### 2.1. Clinical Findings

On initial physical examination, the patient was afebrile, mildly tachypneic, and in stable hemodynamic condition. Breath sounds were markedly diminished over the left lower hemithorax. Cardiac examination revealed a regular rhythm with left bundle branch block on electrocardiography and an ejection fraction according to Simpson of 45–48% by echocardiography. No peripheral edema, neurological deficit, or signs of systemic infection were noted.

### 2.2. Diagnostic Assessment

Chest radiography revealed a large homogeneous opacity occupying the lower two-thirds of the left lung field, consistent with a pleural effusion. Contrast-enhanced computed tomography (CT) of the thorax demonstrated an encapsulated left pleural effusion with adjacent pleural thickening, pleural-pericardial adhesions, and bilateral structural lung deformation due to fibrosis and bronchiectasis. No mediastinal lymphadenopathy or discrete pulmonary nodules were identified. Given the chronicity of the effusion and radiologic suspicion of malignancy, the patient underwent video-assisted thoracoscopic surgery (VATS) on 26 September 2024, including pleural biopsy, pleurodesis, and drainage of 1000 mL of serous pleural fluid. The postoperative course was uneventful.

**Figure 2:**
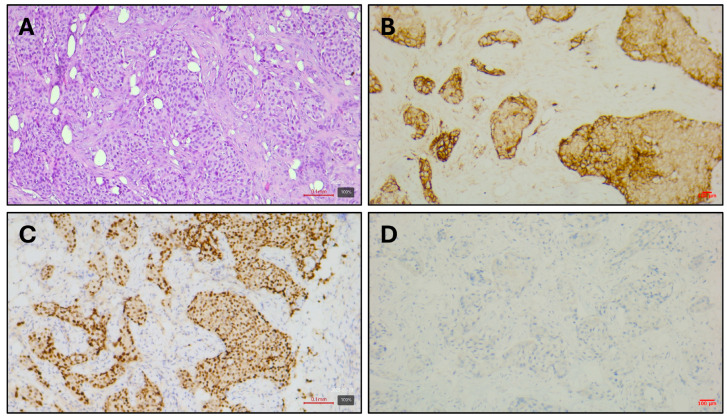
(**A**). H&E of the parietal pleura sample at magnification ×100. (**B**). IHC of PDL-1 using DAKO clone 22C3, ready-to-use antibody at magnification ×100. (**C**). IHC of TTF1 using Dako/Agilent, clone 8G7G3/1, ready-to-use antibody at magnification ×100. (**D**). IHC of ALK using VENTANA clone D5F3, ready-to-use antibody at magnification ×100.

Histopathological examination of the parietal pleura confirmed metastatic infiltration by lung adenocarcinoma (Figure 2A). Immunohistochemistry (IHC) demonstrated strong nuclear TTF-1 positivity, consistent with pulmonary adenocarcinoma origin (Figure 2C). ALK IHC was negative (Figure 2D), and the PD-L1 tumor proportion score (TPS) was 30% based on membranous staining (Figure 2B).

Genomic DNA was extracted from formalin-fixed paraffin-embedded (FFPE) tumor tissue using the AllPrep^®^ DNA/RNA FFPE Kit (Qiagen, Hilden, Germany) following the manufacturer’s instructions. Approximately 120 mm^2^ of FFPE tissue containing ≥30% nucleated tumor cells was selected for analysis. DNA concentration was quantified with a Qubit 4 Fluorometer (Thermo Fisher Scientific, Waltham, MA, USA). Next-generation sequencing (NGS) was performed using the TruSight^®^ Tumor 15 panel (Illumina, San Diego, CA, USA) targeting 15 genes frequently mutated in solid tumors. A total input of 20 ng genomic DNA was used. Target regions were amplified with tagged oligonucleotide primers, and the resulting libraries were indexed and further amplified. Sequencing was conducted on a MiSeqDx platform (Illumina, San Diego, CA, USA). Data analysis was performed using the manufacturer’s analysis module (Illumina, San Diego, CA, USA), applying stringent filters to ensure reliable mutation calling: (i) allelic frequency ≥ 5% and (ii) variant read depth ≥ 500×. Molecular profiling of the tumor revealed an *EGFR* exon 19 deletion along with a baseline *EGFR* exon 20 T790M mutation, both detected prior to initiation of systemic therapy (Table 1). No additional actionable alterations were identified.

**Figure 3:**
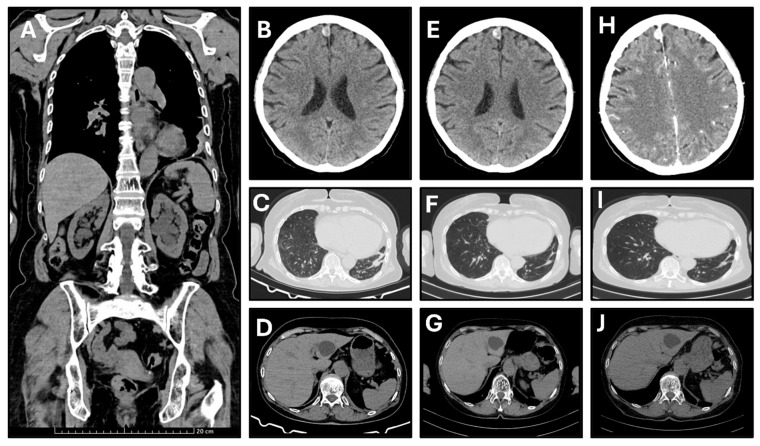
(**A**). Baseline coronal CT scan obtained on 31 October 2024. (**B**–**D**). First restaging axial CT scans performed on 31 January 2025, after 3 months of treatment. (**E**–**G**). Second restaging axial CT scans performed on 24 September 2025, after 9 months of treatment. (**H**–**J**). Third restaging axial CT scans performed on 19 January 2026, after 12 months of treatment.

Staging was completed via total body CT, which was performed on 31 October 2024 (Figure 3A). The disease was staged as cT4N0M1a due to the presence of a malignant left-sided pleural effusion and malignant pleural–pericardial adhesions.

### 2.3. Therapeutic Intervention

Given the patient’s molecular profile, advanced age, comorbidities (ASA IV), and advanced disease, first-line targeted therapy with single-agent osimertinib (80 mg once daily) was recommended. Treatment was initiated on 28 November 2024, in combination with denosumab 120 mg SC monthly for the first six months, followed by every three months thereafter.

### 2.4. Follow-Up and Outcomes

The primary clinical outcome was duration of disease control, defined as the time from initiation of first-line osimertinib to radiologically confirmed disease progression or last follow-up. To enhance clarity and reproducibility in this descriptive case report, radiologic response and disease control were assessed according to RECIST version 1.1, while treatment tolerability and functional status were evaluated using CTCAE version 5.0 and ECOG performance status, respectively. The patient underwent structured clinical, laboratory, and radiologic follow-up (Figure 3B–J), including monthly laboratory assessments and contrast-enhanced CT imaging every 3–6 months. Baseline CT was used as the reference for the RECIST 1.1 assessment. Target lesion selection was performed by the multidisciplinary team and included the primary left basal pulmonary lesion with pleural infiltration, while non-target disease comprised malignant pleural involvement, including prior pleural–pericardial adhesions and effusion.

At the time of data cutoff, the primary outcome was ongoing, exceeding 9 months from initiation of therapy. Best overall response (BOR) was stable disease (SD) per RECIST 1.1. Serial CT evaluations performed in January and September 2025, and January 2026, demonstrated no evidence of progressive disease, with no new visceral, pleural, nodal, cerebral, or osseous lesions identified. The target pulmonary lesion showed no significant change in size, consistent with non-progressive disease, and no recurrence of pleural effusion was observed.

Clinical benefit was accompanied by rapid functional improvement, with ECOG performance status improving from 2 to 0 shortly after treatment initiation and remaining stable throughout follow-up with improved patient-reported quality of life. Treatment was well tolerated, with no treatment-related adverse events observed according to CTCAE version 5.0, and no dose reductions, interruptions, or discontinuations were required. Intracranial findings remained stable. The right frontal extra-axial lesion, radiologically consistent with a meningioma, showed no interval change and no features suggestive of metastatic disease, fulfilling criteria for non-target lesion stability. Overall, these findings demonstrate durable RECIST-defined disease control with sustained clinical benefit and excellent tolerability under first-line osimertinib (Table 2).

## 3. Discussion

This case provides clinically relevant insight into the management of treatment-naïve advanced NSCLC harboring concurrent *EGFR* exon 19 deletion and de novo *EGFR* T790M mutation. The durable RECIST-defined disease control achieved with first-line osimertinib monotherapy in an elderly, multimorbid patient contributes meaningful real-world evidence in a clinical setting where published data remain limited.

The *EGFR T790M* mutation most commonly arises as an acquired resistance mechanism following exposure to earlier-generation TKIs; however, baseline detection has been reported in approximately 0.5–2% of *EGFR*-mutant NSCLC cases using conventional molecular assays, with higher rates observed when highly sensitive techniques are employed [17]. De novo T790M mutations almost invariably coexist with a sensitizing *EGFR* alteration—most frequently exon 21 L858R and less commonly exon 19 deletion, as in the present case [18]. Mechanistically, the T790M substitution increases ATP affinity within the kinase domain, diminishing the inhibitory activity of reversible TKIs and conferring primary resistance to agents such as gefitinib and erlotinib [19].

Historically, pretreatment T790M positivity has been associated with inferior clinical outcomes, including shorter progression-free and overall survival when treated with first-generation TKIs, leading to its characterization as a negative prognostic biomarker [20]. The development of third-generation TKIs, particularly osimertinib, has substantially altered this paradigm. Osimertinib irreversibly inhibits both activating *EGFR* mutations and T790M while sparing wild-type *EGFR*, resulting in improved efficacy and a favorable safety profile [21]. Although pivotal trials such as AURA3 focused primarily on acquired T790M-mediated resistance, accumulating retrospective series and case reports suggest that patients with baseline T790M may also derive clinically meaningful benefit from upfront osimertinib [14].

From a therapeutic standpoint, current NCCN guidelines endorse osimertinib monotherapy, osimertinib combined with platinum–pemetrexed chemotherapy, and lazertinib plus amivantamab as preferred first-line options for patients with *EGFR*-mutated (exon 19 deletion or L858R) advanced nonsquamous NSCLC [22]. The phase III FLAURA2 trial demonstrated superior progression-free and overall survival with the addition of platinum–pemetrexed chemotherapy to osimertinib compared with monotherapy; however, this benefit was accompanied by substantially increased toxicity, with grade ≥3 adverse events reported in approximately 70% of patients receiving combination therapy versus 34% with osimertinib alone [23]. In elderly or multimorbid patients, such toxicity—particularly myelosuppression, fatigue, and infection risk—may significantly impair functional independence and quality of life, potentially offsetting gains in survival [24].

In the present case, initiation of single-agent osimertinib reflected a personalized, patient-centered treatment strategy that prioritized tolerability, preservation of quality of life, and maintenance of functional status over maximal oncologic intensity. Given the patient’s advanced age, significant cardiopulmonary comorbidities, post-COVID pulmonary fibrosis, and absence of rapidly progressive or bulky disease, upfront chemotherapy intensification was considered disproportionate to the anticipated clinical benefit. Notably, osimertinib monotherapy resulted in durable RECIST-defined disease stabilization, rapid improvement in performance status, and excellent long-term tolerability, supporting the appropriateness of this approach.

Several additional aspects merit attention. Despite advanced age and substantial cardiopulmonary comorbidity, the patient tolerated full-dose osimertinib without dose reductions or clinically significant toxicity. Serial contrast-enhanced CT restaging over nine months demonstrated persistent stable disease without the emergence of new visceral, pleural, nodal, cerebral, or osseous metastases, underscoring that de novo T790M does not uniformly confer aggressive tumor biology when effective targeted therapy is administered [25].

The coexistence of moderate PD-L1 expression (TPS 30%) further highlights the importance of molecular context in therapeutic decision-making. Although PD-L1 expression may suggest potential sensitivity to immune checkpoint inhibition in other settings [26,27], *EGFR*-mutant NSCLC has consistently demonstrated limited benefit from immunotherapy, particularly in the absence of prior TKI failure [28,29]. Accordingly, targeted therapy remained the preferred first-line strategy, in line with international recommendations. Additionally, the stable right frontal extra-axial lesion identified on imaging, radiologically consistent with a meningioma, emphasizes the necessity of careful distinction between incidental findings and metastatic disease during response assessment.

Several limitations of this report should be acknowledged. As a single-patient observation, the findings cannot be generalized or used to infer comparative efficacy between therapeutic strategies. The duration of follow-up, while demonstrating durable RECIST-defined disease stabilization exceeding nine months, remains insufficient to assess long-term progression-free or overall survival outcomes. Finally, quantitative assessment of *EGFR T790M* allelic fraction was not available, precluding evaluation of its clonal dominance or potential prognostic impact. In addition, germline testing for *EGFR T790M* was not performed, although the clinical presentation and advanced age make germline origin unlikely.

Despite these limitations, this case highlights broader considerations regarding molecular testing strategies. Baseline assessment for *EGFR T790M* is not routinely performed in all treatment-naïve patients, particularly in resource-limited settings. However, identification of de novo T790M carries direct therapeutic implications, supporting upfront use of third-generation EGFR TKIs and avoiding ineffective exposure to earlier agents. Expanded access to comprehensive *EGFR* genotyping may therefore enhance precision treatment selection, even within rare molecular subgroups.

## 4. Conclusions

This case suggests that de novo *EGFR T790M*–positive NSCLC may represent a biologically and clinically distinct subgroup that can derive meaningful and durable benefit from upfront third-generation EGFR TKI therapy, despite advanced patient age. Optimal management of patients with primary T790M requires a comprehensive multidisciplinary approach integrating molecular diagnostics, comorbidity assessment, geriatric considerations, and patient preferences. Such individualized decision-making enables delivery of effective therapy while maintaining quality of life, even in complex and elderly patient populations.

## Figures and Tables

**Table 1:** Characteristics of the *EGFR* variants detected using next-generation sequencing.

Gene	Variant of Interest	Coordinate	Nucleotide Change	Consequence	Frequency	Coverage
*EGFR*	p.Thr790Met	Chr7:55249071	c.2369C > T	Missense variant	0.210	11,019
*EGFR*	p.Leu747_ Thr751del	Chr7: 55242467	c.2240_ 2254del	Inframe deletion	0.252	405

**Table 2:** Summary of serial CT restaging findings during first-line osimertinib treatment.

Date of CT	Chest (Primary Lesion/Pleura/Lymph Nodes)	Abdomen and Pelvis	Central Nervous System	Bone Assessment	Overall Radiologic Assessment
31 January 2025	Left basal pulmonary lesion without interval change; no pleural effusion; no mediastinal lymphadenopathy	No visceral metastases; stable simple hepatic cyst (35 × 31 mm); no pathological abdominal or pelvic lymphadenopathy	Right frontal parafalcine hyperdense lesion (11 × 8 mm), non-enhancing, suggestive of meningioma; no brain metastases	No CT evidence of progressing osseous metastatic disease	Stable disease; no evidence of progression
24 September 2025	Left basal pulmonary lesion stable; no pleural effusion; no mediastinal lymphadenopathy	No visceral metastases; stable hepatic cyst (34 × 31 mm); no pathological lymph nodes	Right frontal parafalcine lesion unchanged (11 × 8 mm), consistent with meningioma	No CT evidence of progressing osseous metastatic disease	Stable disease; ongoing disease control
19 January 2026	Left basal pulmonary lesion stable; no pleural effusion; no mediastinal lymphadenopathy	No visceral metastases; stable hepatic cyst (37 × 29 mm); no pathological lymph nodes	Right frontal parafalcine lesion unchanged (11 × 8 mm), non-enhancing	No CT evidence of progressing osseous metastatic disease	Stable disease; durable response maintained

Abbreviations: CT: Computer tomography.

## Data Availability

The dataset presented in this article was obtained during routine clinical patient care and is not readily available because of patient privacy protection. However, the data are available from the corresponding author upon reasonable request.

## Supplementary Materials

The following supporting information can be downloaded at: https://www.mdpi.com/article/10.3390/curroncol33050244/s1, Table S1: Baseline Hematologic Parameters; Table S2: Baseline Coagulation Profile; Table S3: Baseline Blood Gas Analysis; Table S4: Baseline Hepatic Function; Table S5: Baseline Renal Function & Metabolic Panel; Table S6: Cardiac Biomarkers (Serial Monitoring); Table S7: Baseline Urinalysis.
