# Supplementary material for: A Case of Primary EGFR T790M Mutation in Treatment-Naïve Advanced NSCLC: Clinical and Molecular Implications
Source: Curr Oncol. 2026 Apr 24;33(5):244. doi: 10.3390/curroncol33050244 (PMC13206194; doi:10.3390/curroncol33050244)
Supplement: Supplementary file 1 [file curroncol-33-00244-s001.zip › curroncol-4212471-supplementary.pdf]

Supplementary Tables: Baseline laboratory values (25 September 2024)

Table S1: Baseline Hematologic Parameters

Parameter	Result	Units	Reference Range	Interpretation
White blood cells (WBC)	9.8	$\times 10^9$/L	3.5–10	Normal
Neutrophils (absolute)	7.8	$\times 10^9$/L	1.6–7.2	Mild elevation
Lymphocytes (absolute)	1.6	$\times 10^9$/L	0.9–2.9	Normal
Mixed cells (MXD#)	0.4	$\times 10^9$/L	0.1–1.5	Normal
Neutrophils (%)	78.9	%	50–70	Elevated
Lymphocytes (%)	16.2	%	22–40	Reduced
Hemoglobin	136	g/L	120–160	Normal
Red blood cells (RBC)	4.32	$\times 10^{12}$/L	4.0–5.4	Normal
Hematocrit	0.41	L/L	0.35–0.50	Normal
MCV	94.3	fL	80–97	Normocytic
MCH	31.6	pg	26.5–33.5	Normal
MCHC	334	g/L	315–350	Normal
RDW-CV	13.6	%	11–16	Normal
Platelets	306	$\times 10^9$/L	150–390	Normal
MPV	8.2	fL	6.5–12	Normal
PDW	10.5	fL	7–15.5	Normal
Plateletcrit	0.25	L/L	Normal	Normal

Table S2: Baseline Coagulation Profile

Parameter	Result	Units	Reference Range	Interpretation
INR	1.12	—	~0.8–1.2	Normal
Prothrombin time	12.5	s	10–15	Normal
Prothrombin activity	88.2	%	70–120	Normal
APTT	34	s	25–38	Normal
Fibrinogen	5.04	g/L	2–4.5	Mild elevation

Table S3: Baseline Blood Gas Analysis

Parameter	Result	Units	Reference Range	Interpretation
pH	7.384	—	7.35–7.45	Normal
pCO_2	38.4	mmHg	35–45	Normal
pO_2	68	mmHg	>80	Mild hypoxemia
HCO_3^-	23.1	mmol/L	23–30	Normal
TCO_2	23.5	mmol/L	—	Acceptable
O_2 saturation	93.8	%	95–100	Mildly reduced
Base excess	−3.1	—	−2 to +2	Mild deficit

Table S4: Baseline Hepatic Function

Parameter	Result	Units	Reference Range	Interpretation
AST (ASAT)	24.0	U/L	0–35	Normal
ALT (ALAT)	20.7	U/L	0–35	Normal
GGT	23.0	U/L	0–38	Normal
Alkaline phosphatase	104	U/L	30–120	Normal
Total bilirubin	9.2	µmol/L	5–21	Normal
Direct bilirubin	2.2	µmol/L	0–6.8	Normal
Total protein	64.1	g/L	66–83	Slightly low
Albumin	38.6	g/L	35–52	Normal

Table S5: Baseline Renal Function & Metabolic Panel

Parameter	Result	Units	Reference Range	Interpretation
Urea	7.2	mmol/L	2.8–7.2	Upper normal
Creatinine	62	µmol/L	44–97	Normal
Glucose	5.03	mmol/L	3.3–6.1	Normal
Sodium	144	mmol/L	135–155	Normal
Potassium	4.6	mmol/L	3.5–5.6	Normal
Alpha-amylase	70	U/L	28–100	Normal

Table S6: Cardiac Biomarkers (Serial Monitoring)

Parameter	Baseline	Follow-up	Units	Reference Range	Interpretation
Creatine kinase (CK)	89.0	83.0	U/L	0–145	Stable
CK-MB	10.5	9.4	U/L	0–24	Stable
Troponin I	0.012	0.014	ng/mL	0–0.06	No myocardial injury

Table S7: Baseline Urinalysis

Parameter	Result	Interpretation
Protein	Negative	Normal
Glucose	Negative	Normal
Ketones	Negative	Normal
Bilirubin	Negative	Normal
Blood	Negative	Normal
Nitrites	Negative	Normal
Specific gravity	1.010	Normal
pH	5.0	Physiologic
Sediment	No pathological findings	Normal
